# Effects of Thermal Activation on CNT Nanocomposite Electrical Conductivity and Rheology

**DOI:** 10.3390/polym14051003

**Published:** 2022-03-02

**Authors:** Joel Hubbard, Joaquin Tirano, Hugo Zea, Claudia Luhrs

**Affiliations:** 1 Mechanical and Aerospace Engineering Department, Naval Postgraduate School, Monterey, CA 93943, USA; ccluhrs@nps.edu; 2 Departamento de Ingeniería Química y Ambiental, Universidad Nacional de Colombia, Bogotá 111321, Colombia; jetiranov@unal.edu.co (J.T.); hrzear@unal.edu.co (H.Z.)

**Keywords:** CNT composites, viscosity, electrically conductive, thermal activation, rheology

## Abstract

Carbon-based nanocomposites featuring enhanced electrical properties have seen increased adoption in applications involving electromagnetic interference shielding and electrostatic dissipation. As the commercialization of these materials grows, a thorough understanding of how thermal activation affects the rheology and electrical performance of CNT–epoxy blends can inform quality decisions throughout the production process. The aim of this work was the identification of the effects that thermal activation has on the electrical and rheological properties of uncured epoxy mixtures and how those may be tied to the resulting cured composites. Herein, three distinct CNT-loaded composite mixtures were characterized for changes in electrical resistivity and viscosity resulting from varying activation times. Electrical conductivity decreased as activation time increased. Uncured mixture viscosity exhibited a strong dependence on CNT loading and applied strain, with activation time being found to significantly reduce the viscosity of the uncured mixture and surface profile of cured composite films. In all cases, cured composites featured improved electrical conductivity over the uncured mixtures. Factors contributing to the observed behavior are discussed. Raman analysis, optical microscopy of CNT networks, and data from silica bead mixing and dispersion studies are presented to contextualize the results.

## 1. Introduction

Many industries are now utilizing nanocomposites due to the enhanced material properties achieved with relatively low nanofiller loadings. One such nanocomposite, carbon nanotube (CNT) epoxy composites, is particularly attractive to the aerospace industry, where favorable electrical properties can be incorporated into structural and adhesive components. CNT’s high aspect ratios enable the generation of electrically conductive composites at extremely low loadings [1,2,3,4]. The reduced resistivity of these materials makes them appealing to a wide variety of industries where electrostatic dissipation (ESD) or electromagnetic interference (EMI) solutions are needed [5,6,7,8,9,10].

The conductivity of the finished material is primarily a function of nanofiller loading and its dispersion within the surrounding matrix.. Inconsistent dispersion resulting in agglomerated areas of CNT bundles or areas devoid of CNTs can occur during the mixing and stages of production. The resulting inhomogeneity in these localized areas can have detrimental effects on the material, potentially causing unreliable performance, hotspots, or premature failure.

Efforts to validate complete CNT dispersion in the finished composite product have employed various techniques. Several groups utilized scanning electron microscopy (SEM) employing voltage or charge contrast imaging to identify CNT bundles within a composite but are limited in practicality for commercial use due to the small sample area and destructive nature of the sample preparation for analysis [11,12,13,14,15]. In [16] rare-earth oxides are utilized to increase the contrast of CNT microstructure in SEM and micro-computed tomography images, and a novel technique utilizing UV-florescence properties of the synthesized nanoparticles is proposed. Pantano et al. [17] proposed infrared imaging as a technique to identify defective areas but could potentially be limited by resolution and analysis depth. In all cases, the finished nanocomposite is inspected after fabrication is completed and the dispersion has already been established, successfully or not. 

Functionalization of the outer CNT surface has been explored as a method to increase solubility or interaction between the CNT and dispersing medium, e.g., solvent or polymer. Approaches typically involve the covalent or noncovalent attachment of chemical groups meant to interact with the matrix material or through the reduction of the CNT surface energy and van der Waals forces [18]. Methods vary widely but have included the attachment of chemical groups such as aminophenyl (C_6_H_4_NH_2_) and nitrophenyl (C_6_H_4_NH_2_) for use in a liquid crystalline polymer [19], polymer functionalization through styryl-grafted MWCNTS [20], and metal or rare-earth oxide attachment [16,21,22]. More straightforward methods of thermal activation or annealing at low temperatures in an oxygen atmosphere have been used to reduce the amount of amorphous carbon, produce defect sites, and increase oxygen-containing functional groups on the CNT surface—resulting in hydrophilic behavior and enhanced dispersion in organic solvents [23,24,25,26]. Overall, surface functionalization can be tailored to the application and can lead to improvements in thermal, mechanical, and electrical properties of the resulting composites by improved dispersion and interaction with the surrounding medium.

Additionally, increased nanofiller loading or surface functionalization causes rheological changes in CNT polymeric mixtures. The correlation of mixture viscosity to the dispersion CNT filler was first documented in [27] and found to vary by CNT wt%. The rheological behavior of CNT–polymer composites, e.g., polycarbonate, polyethylene, and various others [28], as well as epoxy resin composites [29,30,31] have been characterized. The focus has often been centered on changes in the viscosity of the solution or melt mixture the CNTs are added to. The effects of utilizing viscosity-reducing agents have also been explored [32].

To our knowledge, characterization of the effects of thermal activation/annealing on the electrical and rheological properties of uncured epoxy mixtures and the resulting cured composite have not been conducted. The study described in the next sections of this manuscript expands on the existing body of knowledge by characterizing the effects varying activation time has on the electrical conductivity and rheology of an uncured CNT–epoxy mixture, cured composites electrical resistivity, as well as the resulting changes to the phases present and the CNT microstructure. As the use of nanocomposite technologies mature and expand, the need for quality control measures becomes increasingly important to ensure product consistency. The information contained in this study expands the understanding of how production techniques affect end-product performance and can enable the development of other paths for assessing composite properties prior to curing.

## 2. Materials and Methods

### 2.1. Nanofiller and Matrix Materials

Composite synthesis was conducted with unactivated and activated CNT pulp. The CNT pulp was provided by Nanocomp Technologies Inc. (Huntsman Corporation, Merrimack, NH, USA). The multiwall carbon nanotubes (MWCNTs) received were grown as a sheet, produced from nanoparticles of iron that served as catalyst in a chemical vapor deposition process. The resulting product was then reduced to a pulp using an industrial burr mill and holland beater resulting in intertwined bundles approximately 1 mm in length and 0.05 mm in diameter [33] with an iron content of approximately 25.6 wt% [16]. This corresponds to a bundle aspect ratio of ~20. Aspect ratios for individual CNT are considerably higher given that CNT diameter is orders of magnitude smaller than the bundles they comprise.

The unactivated pulp was subjected to an activation process through heating at 500 °C in an open tube furnace (Thermo Scientific Lindberg/Blue M model TF55035A-1, Waltham, MA, USA) for 1, 2, or 2.5 h. Composite samples (cured and uncured) were fabricated with unactivated, and 2.5 h activated CNTs for 0.014 and 0.2 wt% CNT loadings. Composites with 0.75 wt% loading were analyzed in an unactivated state and with activation times of 1, 2, and 2.5 h.

The matrix of the fabricated nanocomposites consisted of Loctite’s EA9396 two-part aerospace epoxy (Henkel Corporation, Dusseldorf, Germany). The cured epoxy has a reported electrical resistivity of 2.14 × 10^15^ Ohm-cm [34].

### 2.2. Sample Synthesis

Unactivated and activated CNTs were dispersed in the epoxy matrix using a FlackTek asymmetric speed mixer (Landrum, SC, USA). Five dispersion cycles were utilized with varying speeds and are featured in Table 1. Low vacuum was applied for 3 min between each dispersion cycle to reduce air entrapment.

Both cured and uncured samples were analyzed in this study. Samples were synthesized using a ratio of 100:30 by weight Part A to Part B and subsequently cured at 66 °C for 1 h. Uncured samples, used for resistivity measurements, and all viscosity determinations, were created without adding the corresponding amount of hardening agent, Part B, or subjecting to elevated temperatures.

### 2.3. Resistivity Measurements

Resistivity was measured directly from prefabricated circuit boards. The boards allow for eight individual measurements of the subject material. The experimental setup and corresponding equations can be found in [16]. Samples were tested without Part B in an uncured state through the application and leveling of a thin film on the circuit board. Cured samples were measured after undergoing the curing process already described. The voltage potential across a 1 cm × 1 cm sample area was measured with a generic digital multimeter under a 5 µA current supplied by a 2400 Keithley source meter (Tektronix, Inc., Beaverton, OR, USA). The epoxy film thickness of cured samples was determined by cross-sectioning and examination via a Nikon Epiphot 200 metallurgical microscope (Nikon, Tokyo, Japan). Uncured sample thickness is approximately the same as the thickness of the tape used to stencil the sample area (0.19 mm).

### 2.4. Sample Characterization

Analysis of the crystalline phases present in the unactivated pulp and at various levels of activation were resolved via X-ray diffraction analysis (XRD) with a Rigaku Miniflex 600 (Woodlands, TX, USA) operating with a Cu source.

CNT bundle characteristics of cured composites were obtained via a metallurgical microscope Nikon Epiphot 200 (Melville, NY, USA). Samples were polished progressively to a 4000 grit and then imaged at 50× magnification.

Raman analysis of CNT pulp samples was conducted using a Renishaw In-via Confocal Raman Microscope (West Dundee, IL, USA). The sample was focused at 50× magnification before being excited with a 633 nm HeNe source utilized in conjunction with 1800 l/mm grating. The data presented represent the average of four locations for each sample.

Viscosity measurements were determined by a Bohlin C-VOR Rheometer acquired from Malvern Instruments Worcestershire, UK. The data were collected at 25 °C using a parallel plate configuration. The diameter of the plate was 20 mm with a gap of 0.5 mm. Data were recorded in oscillatory mode at a fixed frequency of 1 Hz between strains of 1 and 1000%.

## 3. Results and Discussion

### 3.1. Activation Effects on Sample Mass and Phases Present

**Figure 1:**
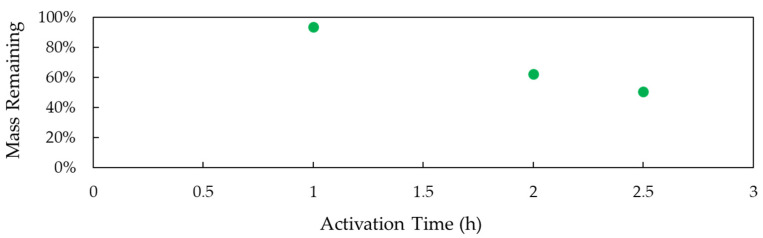
The percentage of remaining mass as a function of activation time illustrates a trend of increasing mass loss as activation time increases.

Thermal activation of the CNT pulp at elevated temperatures results in mass loss due to the oxidation of surface carbon to form CO and CO_2_. Figure 1 presents the mass of CNT remaining after the sample undergoes the activation process. As shown, 93.66, 62.46, and 50.78% mass remains after 1, 2, and 2.5 h activation, respectively.

Additionally, as C==C bonds are broken, oxygen species are introduced as a form of covalent functionalization to the CNT surface [26]. This observation was confirmed in a study by Chen et al., based on X-ray photoelectron spectroscopy binding energy peaks, were assigned to ether C-O-C and quinone C=O during the analysis of CNT pulp thermally activated for durations of 30–120 min at temperatures of 350–550 °C. Increased temperatures were found to preferentially target quinone group functionalization while duration was found to have a minimal effect [25].

The CNT pulp was not treated/cleaned prior to the incorporation of nanotubes into the epoxy mixture, that is, the iron catalyst leftover from the CVD process employed to fabricate the CNT remains present. This approach emulates the process currently employed by industry; the pulp is dispersed into the epoxy as received without being freed of the catalyst. As a result, the thermal activation, apart from modification of the carbon surface, leads to the oxidation of the remaining iron catalyst. Thus, the mass losses represented in Figure 1, and the properties found and described in the next sections, include both the loss of carbon and the oxidation of iron particulates.

**Figure 2:**
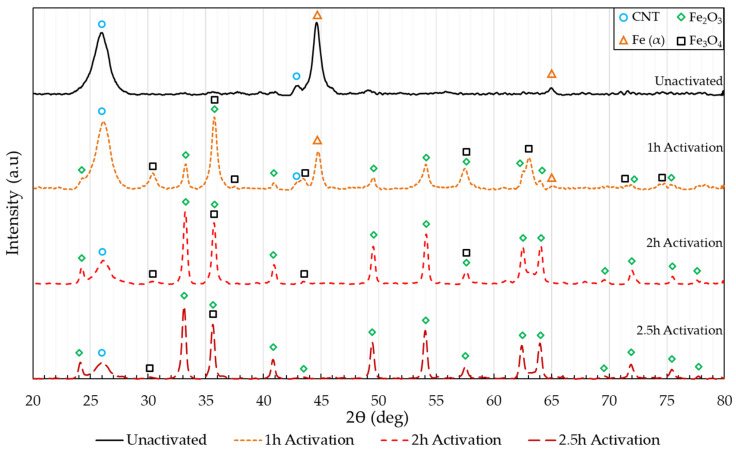
XRD diffraction pattern of unactivated, 1, 2, and 2.5 h activated CNTs and the resulting phase identification.

XRD analysis was performed to determine the effects of thermal activation on crystalline phases in the CNT pulp. The diffraction pattern of the unactivated and activated CNT pulp is revealed in Figure 2. The unactivated CNT pulp consisted of primarily two phases, sp2 carbon arranged as tubular structures composed of rolled-up graphene sheets (CNT), and iron. As the activation time increases, the iron catalysts suffer progressive oxidation; FeO is not detected in the samples activated for 1 h and is likely to only be present only during the initial steps of the reaction, instead, Fe_3_O_4_ and Fe_2_O_3_ peaks are identified. From 1 h to 2 h, the reflections corresponding to metallic iron completely disappear. The reflections of iron (II, III) oxide (Fe_3_O_4_), and iron (III) oxide (Fe_2_O_3_) dominate the pattern, with a marked reduction in the intensity of the peaks corresponding to Fe_3_O_4_ and increase of the Fe_2_O_3_ reflections as the activation time increases. After 2.5 h the iron catalyst has almost fully oxidized to iron (III) oxide.

### 3.2. Electrical Resistivity Characterization

**Figure 3:**
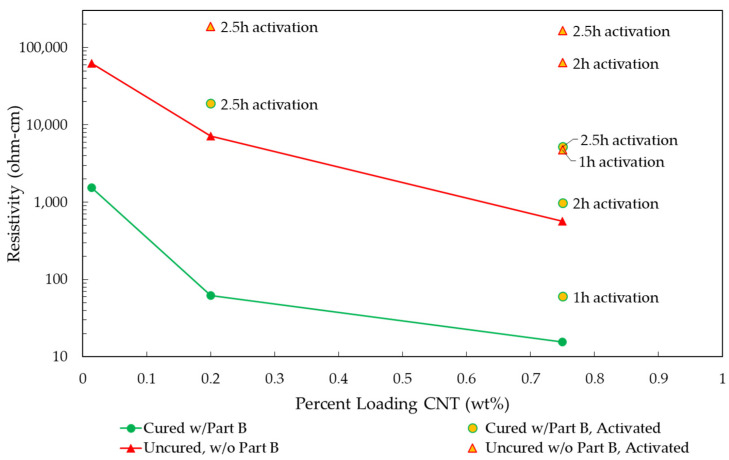
The resistivity of cured and uncured CNT consisting of unactivated and activated CNTs.

The resistivity of 0.014, 0.2, and 0.75 wt% in cured and uncured states with varying levels of activation can be seen in Figure 3. All data points referenced as uncured were measured from a viscous mixture consisting of the epoxy matrix with CNTs dispersed while cured data points come from a solid composite (hardener added to the first mixture and allowed to cure). These loadings were chosen as they span from before to after the percolation limit (which marks the change in conductive mechanisms) is reached [1]. Increased CNT loading resulted in decreased resistivity (increased electrical conductivity) in all cases due to a more robust three-dimensional CNT network being present. Uncured composite mixtures exhibited resistivity higher than the cured composites by factors of 40, 114, and 36 in the 0.014, 0.2, and 0.75 wt% loadings respectively. The 2.5 h activation of 0.014 wt% CNT mixtures resulted in resistivities that exceeded the ability of the test setup to measure accurately. Thermal activation resulted in decreased conductivity in both the uncured mixture and cured composite, the severity of which increased with activation time with a large jump occurring when activation was lengthened from 1 to 2 h. 

One potential cause of this decrease in conductivity is the progressive oxidation of the iron catalyst discussed previously. Iron is traditionally considered a good conductor of electricity. Iron oxides, specifically magnetite (Fe_3_O_4_) and hematite (Fe_2_O_3_), demonstrate more semiconductor qualities depending on particle size and structure [35,36,37,38,39]. It then follows that the large amount of iron initially present (25.6 wt%) transitioning to magnetite (Fe_3_O_4_) and hematite (Fe_2_O_3_) is in part responsible for the increase in resistivity witnessed. The magnitude of which is uncertain without conducting further studies and considering other factors.

Raman analysis found in literature shows that the intensity of the D-band peak at approximately 1350 cm^−1^ increases as MWCNT structural integrity decreases (increased defects), or as the amount of amorphous carbon and impurities increase [40]. Here, it was hypothesized that a decrease in conductivity could be partially attributed to increased defects in the CNT crystalline structure resulting from the thermal activation. This behavior is documented in [25,26], where a study of thermal activation was found to initially reduce amorphous carbon, decreasing D-band intensity. Higher temperatures and longer exposure times eventually overshadowed these effects through defect production resulting in an overall increase of the D-band peak.

**Figure 4:**
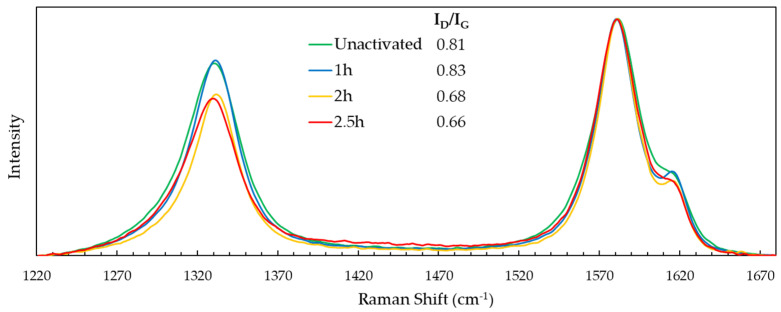
Raman spectroscopy of the D and G-band of unactivated and thermally activated CNT pulp.

Figure 4 presents the results of Raman spectroscopy of unactivated CNT pulp, and that activated for 1, 2, and 2.5 h. The D-band intensity was found to initially increase before decreasing significantly as activation time is increased past one hour. This decrease in the resulting I_D_/I_G_ is indicative of improved CNT quality and a reduction of amorphous carbon—this differs from the overserved changes in peak behavior reported in the previously cited studies by Chen et al. and Mercier et al.

One possible explanation for the difference in D-band behavior when compared to other published work, is that the MWCNT used in this study feature larger aspect ratios and high agglomeration density. The latter is mainly attributed to the fabrication route, which produces intertwined CNT forming a mat, and is later broken into a pulp. As a result, the CNT pulp may experience non-uniform exposure to the activation conditions. The subsequent analysis did reveal D-band intensity varied by location within each individual sample—supporting this conclusion. However, while CNT quality seemingly improved, bulk CNT composite conductivity decreased as activation time increased, making it likely that this impact is minimal and other mechanisms had a more appreciable role in influencing resistivity in the finished composite.

**Figure 5:**
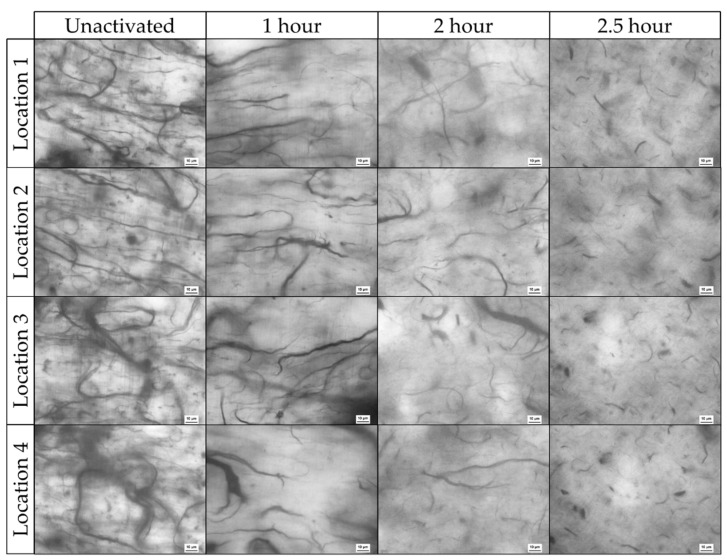
Optical microscopy from four locations in unactivated, 1, 2, and 2.5 h activated CNT pulp. The scale bar is 10 µm with individual panel widths being 143 µm.

Figure 5 presents optical microscopy of finished (cured) nanocomposites produced from CNT that were subjected to varying activation times. It is important to note that samples were fabricated from distinct batches of CNT pulp that were activated for different activation times. The network was imaged in four random locations for each sample. The figure is best observed left to right with the understanding that activation time increases in this direction. An effort was made to keep the focus plane near the middle of the film. 

Initially, a robust CNT network consisting of thick intertwined CNT bundles exists with the bundle lengths spanning most of the field of view, 143 µm wide. Because these structures are not straight and the strands curve through the captured frame, the actual length is assumed to be >143 µm in many locations for both the unactivated and 1 h activated samples. As activation time is increased, a distinct breakdown in the CNT bundle length, bundle diameter, and level of entanglement was observed. Differences between unactivated and 1 h activation times, while discernable, were the least severe, with bundle thickness and length largely intact. Samples fabricated from 2 h activated CNT exhibit a more obvious reduction in bundle thickness and bundle length dropped below 100 µm. Lastly, specimens made with 2.5 h activated pulp show the largest disparity between all the samples with CNT bundle length appearing both more homogenous and significantly shorter, with lengths in the range of10–30 µm. Structural damage to CNT under similar conditions was documented in [25] through TEM imaging. This compromise to the integrity of the CNT itself, with the shear strains of the dispersion process, are the presumed cause of the observed decreases in CNT bundle length.

Past research indicates that the tridimensional CNT network, including the aspect ratio of CNTs, plays a significant role in the conductivity of the resulting composite [41,42,43]. This observed breakdown of the CNT network, to include the length of CNT bundles associated reduction in aspect ratio, is likely a major contributor to the measured increases in resistivity previously discussed.

**Figure 6:**
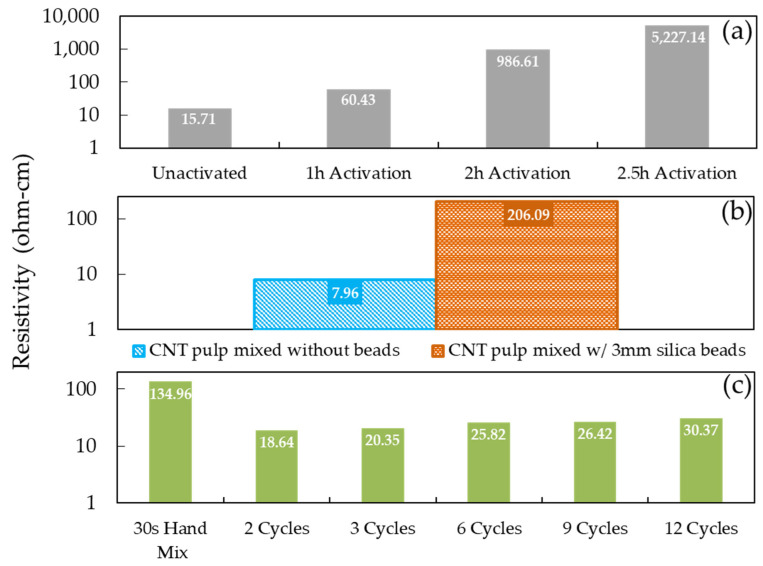
Comparison of 0.75 wt% CNT cured composite resistivities when activation time is varied (**a**), silica beads are used in the mixing process to encourage separation of tube bundles (**b**), and the number of mixing cycles is varied (**c**).

The electrical resistivity measurements obtained in this study by varying activation duration were compared with two datasets obtained in previous studies, (1) varying the number of cycles in the mixing protocol and, (2) utilizing 3 mm silica beads to aid dispersion. Figure 6 presents the 0.75 wt% CNT composite electrical resistivity derived here (Figure 6a) alongside the two datasets adapted from [1] (Figure 6b,c). The silica beads were added prior to the mixing process and strained out of the mixture prior to curing (prior to cycle 1, and after cycle 5 in Table 1) to aid dispersion of the CNT pulp. These beads introduce shear forces that have the effect of increasing dispersion and decreasing bundle size by impact and attrition—essentially functioning as grinding media within the asymmetric mixture. This breakdown of the interconnected CNT network leads to an increase in the resistivity of the composite. Similarly, mixing (without beads) initially improves conductivity through the dispersion of the CNT filler, however, past a certain threshold the resistivity increases as the CNT pulp is over-mixed. While 0.75 wt% CNT loading was analyzed here, note that these effects differ (less severe) at loadings below the percolation limit, e.g., 0.014%. In sum, electrical resistivity depends on the connectedness of the CNT network for a percolation path, past a certain level of dispersion, further homogeneity can result in a reduction of electrical conductivity. For a more in-depth discussion on percolation theory and these mechanisms, the reader is directed to [1].

The magnitude of the effects observed in previous studies, i.e., the use of beads and varying mixing cycles, is significantly smaller than those seen to result from thermal activation (this study). Over-mixing through additional dispersion cycles results in a ~2x increase in resistivity, while bead mixing has a more meaningful impact of 1–2 orders of magnitude. Activation changes result in 2–3 orders of magnitude difference. This suggests that the differences are likely the result of an amalgamation of the changes documented thus far, e.g., of phase changes associated with the remaining iron catalyst oxidation, a decreased interconnected CNT network, and reduced aspect ratio of the CNT pulp, and potentially increased dispersion resulting from surface functionalization.

### 3.3. Viscosity Characterization

**Figure 7:**
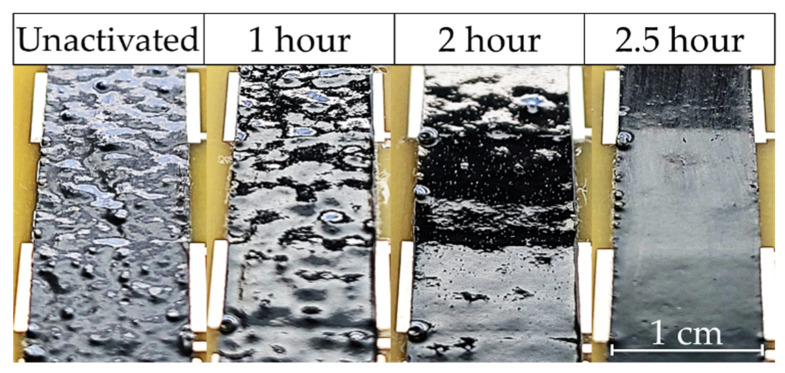
Close-up view of cured epoxy fabricated with CNTs under various levels of activation.

Notable differences in the viscosity of activated CNT/Epoxy mixtures are observed when working with unactivated and activated CNTs within the studied loading (% of CNT) regime. Activated samples tend to agglomerate less and have a more consistent texture, often leading to greater ease of application onto surfaces for thin-film samples production. Indicative of this is the post-cured appearance of ~0.19 mm thin-film samples shown in Figure 7. The finished texture, more specifically the surface profile of the cured composite, changes drastically based on activation level. The unactivated CNT film appears distinctly as a sample with varied heights with activated samples reducing this effect and eventually curing as a smooth surface in the case of 2.5 h activation. When interpreted in conjunction with the breakdown of CNT bundles seen in optical microscopy presented in Figure 5, we speculate that the inconsistencies in profile height are the result of larger agglomerations causing raised areas in the composite. That is, areas higher in profile height are likely dense regions of CNTs. As the CNT network becomes less interconnected as a result of the activation, namely due to bundle length and thickness decreasing as discussed in relation to Figure 5, this effect decreases.

**Figure 8:**
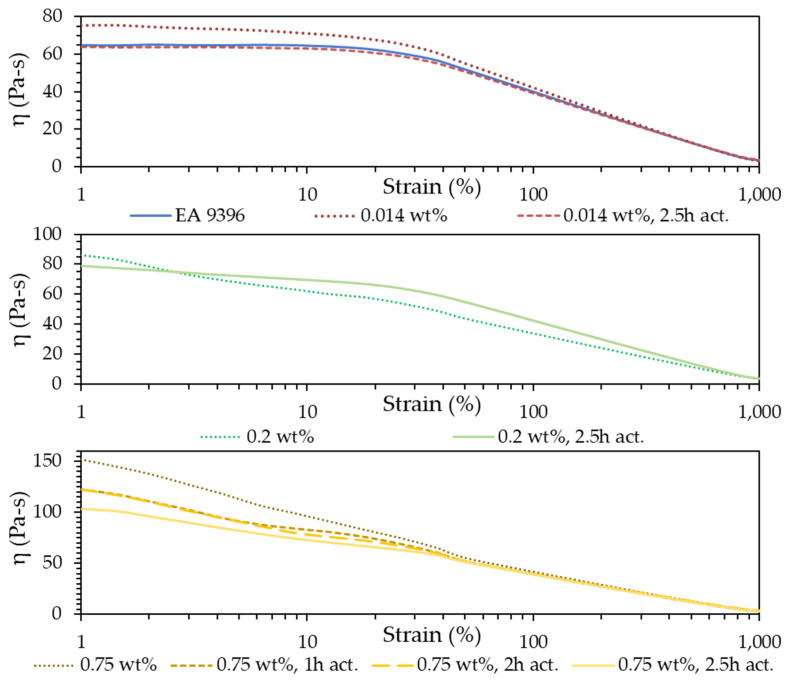
Measured viscosity as a function of strain for 0.014, 0.02, and 0.75 wt% CNT loadings with different activation times.

Figure 8 presents the viscosity measurements obtained from nine unique samples. At low strains, increased CNT loading percentage results in increased viscosity. In all cases, viscosity decreases with increasing strain. Above ~40% strain a distinct change in slope occurs as the samples converge to similar viscosities. Prior to this point, higher CNT loadings produce steeper decreases in viscosity as strain increases. The behavior is consistent with the findings of Rahatekar et al., where through optical analysis and comparable viscosimeter geometry, viscosity at low shear strain rates was found to increase with MWCNT loading and shear thinning behavior was correlated to the breakdown of CNT bundle agglomeration [44]. The convergence of all samples to a similar point indicates that a breakdown of the agglomerations has occurred and past this ~40% strain, viscosity is dominated by the properties of the base epoxy.

Of the nine samples, one exhibits behavior inconsistent with the rest. At low strains, the 0.2 wt% CNT sample presents viscosity consistent with what is to be expected, i.e., greater than the 0.014% loadings and less than the 0.75% loadings. However, it decreases more rapidly as strain increases and does not converge to the same point as the rest. The consistency in the behavior of the rest of the samples leads the authors to speculate that other factors may be at play. The raw CNT pulp is sometimes very tightly agglomerated. If the required loading featured more of these regions than normal, it is possible the resulting dispersion was inconsistent with other samples. Differences in environmental run conditions of the viscosimeter could also lead to differences in behavior. Further testing and analysis of the sample in question would need to be performed to confirm the mechanism for such discrepancy.

Thermal activation had the effect of decreasing the viscosity of the mixture compared to its unactivated counterpart at all strains. The only exception was the outlier already discussed (general trend still held true at low strains). The 0.014 wt% mixtures display a slightly higher viscosity than the base epoxy but, after thermal activation, drops to a level near pure EA9396. At 0.75% loadings, thermal activations effect on decreasing viscosity is also seen which the most dramatic fall coming at 2.5 h activation time. Large differences in the slope at which viscosity decreases are seen for the two loadings. The differences in this initial slope are attributed to large differences in the microstructure. At 0.014%, below the percolation limit, the connectedness of the CNT network is sparse, with CNT bundle interaction having a limited effect on both the initial viscosity on the mixture and resulting in weak dependence on strain. At 0.75%, a robust interconnect network of CNT bundles exists, viscosity at low strains increases dramatically and increasing strain results in larger reductions in viscosity as shear forces separate agglomerates and interactions between CNT bundles.

The effects of CNT functionalization with oxygen species and its impact on viscosity may need further study and have been targeted for future work. The vast differences in the size of the CNT pulp seen in Figure 5 make it difficult to decouple the viscosity effects of shorter CNT bundles from any improvements that functionalization caused through improved matrix interaction. However, the combined result of thermal activation is a significant decrease in mixture viscosity across all loadings.

These results involving the electrical resistivity of uncured epoxy–CNT mixtures and cured epoxy–CNT nanocomposites suggest a predictable relationship between the electrical performance in uncured and cured states at all levels of thermal activation. Rheological measurements also found notable trends within each CNT wt% loading regime where both thermal activation duration and decreased CNT loading result in measurable decreases in viscosity at lower strains. This points to an opportunity to identify poor dispersion, loading, or subpar electrical performance of a resulting nanocomposite through in-line quality control—something readily employed by many industries. Electrical resistivity has been identified for use in the concrete industry for testing mixtures’ resistance to chloride permeability and the associated corrosion of mechanical reinforcements [45,46]. Even more widespread, many industries utilize viscosity as a metric to ensure the quality of the resulting product. The food industry utilizes viscosity to guarantee texture and batch-to-batch consistency [47]. Pharmaceutical manufacturers utilize viscosimeters at regular checkpoints to safeguard solutions, emulsions, and suspensions critical to drug delivery [48,49]. Similarly, coatings and lubricants are subjected to viscosity standards to ensure the proper application method is chosen and desired thickness is achieved [50,51,52], or lubricating protection is maintained [53,54,55]. Findings such as the ones presented in this manuscript make the development of similar techniques utilizing epoxy–CNT mixture conceivable.

## 4. Conclusions

The data collected here indicate that thermal activation has significant impacts on the electrical and rheological performance of CNT–epoxy mixtures both in production and after curing. Thermal activation of CNT pulp increases resistivity by 2–3 orders of magnitud in both uncured and cured CNT–epoxy composites. These effects were documented across loading regimes. The resistivity of uncured mixtures mirrored the behavior of cured CNT composites, suggesting that the absent other defects in the production process the performance of the uncured mixture could be an indicator of finished composite performance. The thermal activation of CNT pulp also dramatically affected the uncured mixtures’ viscosity. Viscosity was found to increase with increasing CNT loading and be inversely dependent on activation time. At high strains, the mixtures viscosity approximates that of the base epoxy.

These findings expand upon the existing body of knowledge already available and demonstrate that there are verified trends within CNT loading regimes as activation time is varied. Additionally, they show the potential for precured viscosity and mixture resistivity as an indicator of the behavior and properties of the corresponding cured composite. When taken together this data has demonstratable value to industry, namely in the form of understanding how the effects of thermal activation, as a functionalization technique, change dispersed CNT mixtures and the corresponding composite performance. As commercialization grows this data can facilitate inspection and validation efforts of CNT–polymer composites at a stage of production that would still allow for remediation.

## Figures and Tables

**Table 1:** Steps of the synthesis process for cured and uncured CNT nanocomposite samples.

		Cycle 1		Cycle 2		Cycle 3		Cycle 4			Cycle 5	
**Cured Samples**	Part A + CNTs Added	Low Speed (1 min)	3 min Vacuum	Medium Speed (2 min)	3 min Vacuum	High Speed (1 min)	3 min Vacuum	High Speed (1 min)	Part B Added	3 min Vacuum	High Speed (1 min)	1 h at 66 °C
**Uncured Samples**												

## References

[1] Earp B., Simpson J., Phillips J., Grbovic D., Vidmar S., McCarthy J., Luhrs C. (2019). Electrically Conductive CNT Composites at Loadings below Theoretical Percolation Values. Nanomaterials.

[2] Sandler J.K.W., Kirk J.E., Kinloch I.A., Shaffer M.S.P., Windle A.H. (2003). Ultra-Low Electrical Percolation Threshold in Carbon-Nanotube-Epoxy Composites. Polymer.

[3] Martin C.A., Sandler J.K.W., Shaffer M.S.P., Schwarz M.-K., Bauhofer W., Schulte K., Windle A.H. (2004). Formation of Percolating Networks in Multi-Wall Carbon-Nanotube–Epoxy Composites. Compos. Sci. Technol..

[4] Sandler J., Shaffer M.S.P., Prasse T., Bauhofer W., Schulte K., Windle A.H. (1999). Development of a Dispersion Process for Carbon Nanotubes in an Epoxy Matrix and the Resulting Electrical Properties. Polymer.

[5] Bellucci S., Balasubramanian C., Micciulla F., Rinaldi G. (2007). CNT Composites for Aerospace Applications. J. Exp. Nanosci..

[6] Du J.-H., Bai J., Cheng H.-M. (2007). The Present Status and Key Problems of Carbon Nanotube Based Polymer Composites. Express Polym. Lett..

[7] Harris P.J.F. (2004). Carbon Nanotube Composites. Int. Mater. Rev..

[8] Kausar A., Rafique I., Muhammad B. (2016). Review of Applications of Polymer/Carbon Nanotubes and Epoxy/CNT Composites. Polym. Plast. Technol. Eng..

[9] Kumar P., Maiti U.N., Sikdar A., Das T.K., Kumar A., Sudarsan V. (2019). Recent Advances in Polymer and Polymer Composites for Electromagnetic Interference Shielding: Review and Future Prospects. Polym. Rev..

[10] Park S.-H., Ha J.-H. (2019). Improved Electromagnetic Interference Shielding Properties Through the Use of Segregate Carbon Nanotube Networks. Materials.

[11] Li W., Buschhorn S.T., Schulte K., Bauhofer W. (2011). The Imaging Mechanism, Imaging Depth, and Parameters Influencing the Visibility of Carbon Nanotubes in a Polymer Matrix Using an SEM. Carbon.

[12] Finnie P., Kaminska K., Homma Y., Austing D.G., Lefebvre J. (2008). Charge Contrast Imaging of Suspended Nanotubes by Scanning Electron Microscopy. Nanotechnology.

[13] Loos J., Alexeev A., Grossiord N., Koning C.E., Regev O. (2005). Visualization of Single-Wall Carbon Nanotube (SWNT) Networks in Conductive Polystyrene Nanocomposites by Charge Contrast Imaging. Ultramicroscopy.

[14] Lillehei P.T., Kim J.-W., Gibbons L.J., Park C. (2009). A Quantitative Assessment of Carbon Nanotube Dispersion in Polymer Matrices. Nanotechnology.

[15] Kovacs J.Z., Andresen K., Pauls J.R., Garcia C.P., Schossig M., Schulte K., Bauhofer W. (2007). Analyzing the Quality of Carbon Nanotube Dispersions in Polymers Using Scanning Electron Microscopy. Carbon.

[16] Hubbard J., Isik T., Ansell T.Y., Ortalan V., Luhrs C. (2021). Introduction of Rare-Earth Oxide Nanoparticles in CNT-Based Nanocomposites for Improved Detection of Underlying CNT Network. Nanomaterials.

[17] Pantano A., Montinaro N., Cerniglia D., Micciulla F., Bistarelli S., Cataldo A., Bellucci S. (2019). Novel Non-Destructive Evaluation Technique for the Detection of Poor Dispersion of Carbon Nanotubes in Nanocomposites. Compos. Part B Eng..

[18] Mallakpour S., Soltanian S. (2016). Surface Functionalization of Carbon Nanotubes: Fabrication and Applications. RSC Adv..

[19] Sahoo N.G., Cheng H.K.F., Bao H., Pan Y., Li L., Chan S.H. (2011). Covalent Functionalization of Carbon Nanotubes for Ultimate Interfacial Adhesion to Liquid Crystalline Polymer. Soft Matter..

[20] Hua J., Wang Z., Xu L., Wang X., Zhao J., Li F. (2013). Preparation Polystyrene/Multiwalled Carbon Nanotubes Nanocomposites by Copolymerization of Styrene and Styryl-Functionalized Multiwalled Carbon Nanotubes. Mater. Chem. Phys..

[21] Xin F., Li L. (2011). Decoration of Carbon Nanotubes with Silver Nanoparticles for Advanced CNT/Polymer Nanocomposites. Compos. Part Appl. Sci. Manuf..

[22] Ma P.C., Tang B.Z., Kim J.-K. (2008). Effect of CNT Decoration with Silver Nanoparticles on Electrical Conductivity of CNT-Polymer Composites. Carbon.

[23] Wang K., Fishman H.A., Dai H., Harris J.S. (2006). Neural Stimulation with a Carbon Nanotube Microelectrode Array. Nano Lett..

[24] Klein K.L., Melechko A.V., McKnight T.E., Retterer S.T., Rack P.D., Fowlkes J.D., Joy D.C., Simpson M.L. (2008). Surface Characterization and Functionalization of Carbon Nanofibers. J. Appl. Phys..

[25] Chen X., Tang X.-Z., Liang Y.N., Cheah J.W., Hu P., Hu X. (2016). Controlled Thermal Functionalization for Dispersion Enhancement of Multi-Wall Carbon Nanotube in Organic Solvents. J. Mater. Sci..

[26] Mercier G., Gleize J., Ghanbaja J., Marêché J.-F., Vigolo B. (2013). Soft Oxidation of Single-Walled Carbon Nanotube Samples. J. Phys. Chem. C.

[27] Huang Y.Y., Ahir S.V., Terentjev E.M. (2006). Dispersion Rheology of Carbon Nanotubes in a Polymer Matrix. Phys. Rev. B.

[28] Arrigo R., Malucelli G. (2020). Rheological Behavior of Polymer/Carbon Nanotube Composites: An Overview. Materials.

[29] Allaoui A., Bounia N. (2010). Rheological and Electrical Transitions in Carbon Nanotube/Epoxy Suspensions. Curr. Nanosci..

[30] Fogel M., Parlevliet P., Geistbeck M., Olivier P., Dantras E. (2015). Thermal, Rheological and Electrical Analysis of MWCNTs/Epoxy Matrices. Compos. Sci. Technol..

[31] Chakraborty A.K., Plyhm T., Barbezat M., Necola A., Terrasi G.P. (2011). Carbon Nanotube (CNT)–Epoxy Nanocomposites: A Systematic Investigation of CNT Dispersion. J. Nanoparticle Res..

[32] Al-Saleh M.H., Irshidat M.R. (2016). Effect of Viscosity Reducing Agent on the Properties of CNT/Epoxy Nanocomposites. J. Polym. Eng..

[33] Menchhofer P.A., Johnson J.E., Lindahl J.M. (2016). Carbon Nanotube Chopped Fiber for Enhanced Properties in Additive Manufacturing.

[34] LOCTITE 9396aero. https://www.henkel-adhesives.com/vn/en/product/adhesives/loctite_ea_9396_aero.html.

[35] Mochizuki S. (1977). Electrical Conductivity of α-Fe_2_O_3_. Phys. Status Solidi A.

[36] Dutta S., Manik S.K., Pal M., Pradhan S.K., Brahma P., Chakravorty D. (2005). Electrical Conductivity in Nanostructured Magnetite–Hematite Composites Produced by Mechanical Milling. J. Magn. Magn. Mater..

[37] Weidenfeller B., Höfer M., Schilling F. (2002). Thermal and Electrical Properties of Magnetite Filled Polymers. Compos. Part Appl. Sci. Manuf..

[38] Hematite. https://phantomplastics.com/functional-fillers/hematite-filler/.

[39] Magnetite. https://phantomplastics.com/functional-fillers/magnetite/.

[40] Osswald S., Flahaut E., Ye H., Gogotsi Y. (2005). Elimination of D-Band in Raman Spectra of Double-Wall Carbon Nanotubes by Oxidation. Chem. Phys. Lett..

[41] Celzard A., McRae E., Deleuze C., Dufort M., Furdin G., Marêché J.F. (1996). Critical Concentration in Percolating Systems Containing a High-Aspect-Ratio Filler. Phys. Rev. B.

[42] Majidian M., Grimaldi C., Forró L., Magrez A. (2017). Role of the Particle Size Polydispersity in the Electrical Conductivity of Carbon Nanotube-Epoxy Composites. Sci. Rep..

[43] Tarlton T., Sullivan E., Brown J., Derosa P.A. (2017). The Role of Agglomeration in the Conductivity of Carbon Nanotube Composites near Percolation. J. Appl. Phys..

[44] Rahatekar S.S., Koziol K.K.K., Butler S.A., Elliott J.A., Shaffer M.S.P., Mackley M.R., Windle A.H. (2006). Optical Microstructure and Viscosity Enhancement for an Epoxy Resin Matrix Containing Multiwall Carbon Nanotubes. J. Rheol..

[45] Rupnow T., Icenogle P. (2012). Surface Resistivity Measurements for Quality Assurance Pave the Way to Savings in Louisiana.

[46] Ferreira R.M., Jalali S. Quality Control Based on Electrical Resistivity Measurements. Proceedings of the European Symposium on Service Life and Serviceability of Concrete Structures.

[47] Importance of Viscosity in Food Manufacturing from Cole-Parmer. https://www.coleparmer.com/tech-article/viscosity-in-food-manufacturing.

[48] Quality Control of Pharmaceutical Products Using Rotational Viscometry: Anton Paar Wiki. https://wiki.anton-paar.com/us-en/basic-of-viscometry/quality-control-of-pharmaceutical-products-using-rotational-viscometry/.

[49] Allen L.V. (2003). Quality-Control Analytical Methods: Viscosity Measurement. Int. J. Pharm. Compd..

[50] Paints and Coatings: Quality Control and Research: Anton-Paar.Com. https://www.anton-paar.com/us-en/viscometry-rheometry/paints-and-coatings-quality-control-and-research/.

[51] Dash M. High Quality ‘Greener’ Coatings through Automated Viscosity Monitoring and Control—Rheonics: Viscometer and Density Meter. https://rheonics.com/high-quality-greener-coatings-through-automated-viscosity-monitoring-and-control/.

[52] Optimizing the Measurement and Control of Viscosity for Consistent Quality-Lubrizol. https://www.lubrizol.com/Coatings/Blog/2021/09/Viscosity-for-Consistent-Quality.

[53] Lubricants|Viscosity Measurement for In-Service Oil: Anton-Paar.Com. https://www.anton-paar.com/corp-en/services-support/document-finder/application-reports/lubricants-viscosity-measurement-for-in-service-oil/.

[54] Viscosity Index: Anton Paar Wiki. https://wiki.anton-paar.com/us-en/viscosity-index/.

[55] Alarms 101: Setting Viscosity Alarms and Limits. https://www.machinerylubrication.com/Read/429/viscosity-alarms-limits.

